# 5‐fluoro‐deoxyglucose PET/CT response after neoadjuvant chemotherapy predicts long‐term outcomes in soft tissue sarcomas: Results from a prospective trial

**DOI:** 10.1002/cncr.70129

**Published:** 2025-10-10

**Authors:** Edward Y. Cheng, Andrea P. Espejo Freire, Juan C. Manivel, Jerry W. Froelich, Brenda J. Weigel, Denis R. Clohisy, Christian M. Ogilvie, Rich Evans, L. Chinsoo Cho, Kathryn E. Dusenbery, Keith M. Skubitz

**Affiliations:** ^1^ Department of Orthopedic Surgery The Masonic Cancer Center The University of Minnesota Medical School University of Minnesota Minneapolis Minnesota USA; ^2^ Department of Medicine The Masonic Cancer Center The University of Minnesota Medical School University of Minnesota Minneapolis Minnesota USA; ^3^ Department of Pathology and Laboratory Medicine Minneapolis Veterans Affairs Medical Center Minneapolis Minnesota USA; ^4^ Department of Radiology The Masonic Cancer Center The University of Minnesota Medical School University of Minnesota Minneapolis Minnesota USA; ^5^ Department of Oncology St Jude Children’s Research Hospital Memphis Tennessee USA; ^6^ Biostatistics Core The Masonic Cancer Center The University of Minnesota Medical School University of Minnesota Minneapolis Minnesota USA; ^7^ Department on Radiation Oncology The Masonic Cancer Center The University of Minnesota Medical School University of Minnesota Minneapolis Minnesota USA

**Keywords:** doxorubicin, ifosfamide, neoadjuvant therapy, positron emission tomography, prognosis, sarcoma, soft tissue

## Abstract

**Background:**

Only 30%–40% of high‐grade sarcomas respond to initial chemotherapy, which can have significant toxicities. Computed tomography imaging alone has limitations in evaluating treatment response. Imaging with 5‐fluoro‐deoxyglucose–positron emission tomography/computed tomography (FDG‐PET/CT), which evaluates tumor metabolism, offers an alternative. This study examined whether early changes in the maximum standardized uptake value (SUVmax) after neoadjuvant chemotherapy can be used to predict outcomes in high‐grade sarcomas.

**Methods:**

This prospective trial assessed whether changes in the SUVmax after one or four cycles of pegylated‐liposomal doxorubicin plus ifosfamide could predict progression‐free survival (PFS), overall survival (OS), and histologic response. Fifty‐six patients were required for 90% power. Metabolic response was defined as a reduction ≥40% in the SUVmax from baseline after either cycle 1 (delta 1) or cycle 4 (delta 2).

**Results:**

Sixty‐nine patients were enrolled (2006–2013) with a median follow‐up of 8.1 years. The 10‐year PFS rate was 74% versus 42% for delta 1 responders versus nonresponders (*p* = .0082), respectively; and 69% versus 33% for delta 2 responders versus nonresponder (*p* = .0015), respectively. The 10‐year OS rate was 73% versus 58% for delta 1 responders versus nonresponder (*p* = .28), respectively; and 81% versus 37% for delta 2 responders versus nonresponder (*p* = .00026), respectively. Of 46 delta 1 nonresponders, 23 met criteria at delta 2. A positron emission tomography response was correlated with histologic necrosis. At last follow‐up, 31 patients (44.9%) were alive and disease free, and 10 (14.5%) were alive with sarcoma. Five patients developed secondary malignancies.

**Conclusions:**

An SUVmax reduction verified on FDG‐PET/CT imaging after neoadjuvant chemotherapy was a strong predictor of long‐term outcomes. Metabolic imaging at treatment completion identified responders, supporting continued therapy in initially nonresponsive patients and guiding personalized treatment strategies.

## Abstract 2

Assessing the response of patients who have high‐grade soft tissue sarcomas after neoadjuvant chemotherapy by using FDG‐PET/CT imaging is strongly associated with long‐term outcomes. The metabolic response at treatment completion could identify patients who are likely to benefit from personalized treatment strategies.

## INTRODUCTION

Managing high‐risk soft tissue sarcomas presents significant challenges. Although evidence has been historically mixed, it supports the use of intense neoadjuvant chemotherapy in selected patients.^1^, ^2^, ^3^, ^4^, ^5^ However, these regimens are highly toxic, and decisions to initiate or continue neoadjuvant chemotherapy can be difficult. Data indicate that changes in tumor size do not reliably reflect the response to treatment. Tumors can be asymmetrical, with varying necrosis at baseline, and some tumors paradoxically enlarge as necrosis progresses. Thus, the Response Evaluation Criteria in Solid Tumors criteria have failed at predicting outcomes in patients with soft tissue and bone sarcomas.^6^, ^7^, ^8^ Incorporating changes in tumor density observed through contrast‐enhanced computed tomography (CT) to reflect necrosis has improved the ability to predict histologic response and disease‐free survival.^9^, ^10^

Functional imaging with 5‐fluoro‐deoxyglucose–positron emission tomography (FDG‐PET) combined with CT offers the advantage of assessing both tumor metabolism and size changes. In 1999, Schulte et al. demonstrated that changes in FDG‐PET uptake correlated with a positive histologic response in 27 patients who had osteosarcoma.^11^ Subsequent studies have demonstrated its utility in diagnosis,^12^, ^13^ staging,^14^ histologic response,^15^, ^16^ and survival prediction in high‐grade soft tissue sarcomas.^13^, ^17^, ^18^, ^19^ However, evidence remains limited, and most studies have been constrained by small size, retrospective design, and focus on early endpoints rather than long‐term outcomes.

Because many patients are older and have multiple comorbidities, early detection of chemotherapy benefit is crucial to avoid unnecessary toxicity. To address this, we prospectively evaluated the role of FDG‐PET scans after one cycle of chemotherapy and upon completion of the neoadjuvant regimen. In this report, we present the 15‐year follow‐up results of a prospective study aimed at determining whether early changes in the maximum standardized uptake value (SUVmax) on FDG‐PET can predict progression‐free survival (PFS) in patients with high‐grade soft tissue sarcomas who received neoadjuvant pegylated‐liposomal doxorubicin (PLD) plus ifosfamide. Secondary end points that we assessed were histologic response and overall survival (OS).

## MATERIALS AND METHODS

In 2006, after receiving approval from the University of Minnesota Institutional Review Board, we began a prospective clinical trial for patients older than 16 years who had high‐grade (French National Federation of Cancer Centers grade 3) soft tissue sarcomas of the extremities or body wall, with tumors larger than 5 cm in greatest dimension. Patients with unresectable tumors at diagnosis were not enrolled. Sixty‐nine patients were enrolled between 2006 and 2014 and continued follow‐up until May 2021. The trial protocol included preoperative chemotherapy, wide surgical excision, and subsequent external‐beam radiation.

### Treatment regimen

All patients received PLD at 45 mg/m^2^ intravenously on day 1 combined with ifosfamide administered by continuous intravenous infusion at 1.5 g/m^2^ daily for 6 days (total dose, 9 g/m^2^) plus mesna at 1.5 g/m^2^ daily for 7 days within 28‐day cycles.^20^ We used granulocyte‐colony–stimulating factor prophylactically in both regimens. Our institution replaced free doxorubicin with PLD because of its significantly reduced toxicity and at least comparable efficacy.^21^, ^22^, ^23^, ^24^

### 5‐fluoro‐deoxyglucose–positron emission tomography

FDG‐PET scans were performed at baseline before chemotherapy, after cycle 1 of chemotherapy, and after cycle 4 of chemotherapy, just before wide surgical excision. Scans were conducted 60–90 minutes after administering 558 ± 48 megabecquerels of FDG, with patients fasting for at least 4 hours. A Siemens Biograph‐16 or mCT‐64 scanner (Siemens Healthineers) equipped with high‐resolution crystals was used. The scanner was cross‐calibrated with the dose calibrator, and clocks were synchronized. The protocol included CT imaging from head to feet, followed by a whole‐body emission PET scan of the same region, with a resolution of 220 × 220. PET and CT images were co‐registered using table positional information, and attenuation corrections were applied based on CT data. PET images were reconstructed using an iterative reconstruction algorithm with ordered‐subset expectation maximization. The SUVmax was calculated by identifying a region of interest that encompassed the gross tumor volume, with the manufacturer's algorithm applied. No corrections for body surface area or patient size were made. Change in the SUVmax (delta SUVmax) was calculated, and the response was measured as a continuous variable or categorized as responder (≥40% reduction in the SUVmax) or nonresponder (<40% reduction in the SUVmax). The FDG‐PET response was evaluated after one cycle of chemotherapy (delta 1) and after the completion of all chemotherapy cycles (delta 2).

### Pathologic analysis

All patients had a histologic diagnostic biopsy before trial enrollment. Wide excision was performed on all patients with localized, resectable disease using standard oncologic techniques to achieve negative surgical margins (R0 resection). A single sarcoma pathologist (J.C.M.) conducted uniform pathologic assessments. The specimen was oriented, surgical margins were marked with ink, and a section was made along the largest axis. Maximum dimensions of the specimen and tumor were measured, and gross photographs of the cut surface were obtained. For histologic examination, a 3‐mm central slice was fixed in 10% buffered formalin, serially sectioned, and submitted fully for analysis. A diagram was constructed to identify each tissue block's source and its relation to gross features. Standard soft tissue sarcoma pathologic processing was followed. Microscopic examination was performed on hematoxylin‐and‐eosin–stained sections using an Olympus BX40 microscope (Olympus Optical Company Ltd.). The parameters evaluated included the distance of the tumor from surgical margins, the percentage of viable tumor, and the percentage of nonviable tumor, addressing necrosis, fibrosis, hemorrhage, and cystic change. Each case received a specific histologic diagnosis and grade according to the National Cancer Institute and French National Federation of Cancer Centers systems.^25^, ^26^ Histologic response was defined as 100% minus the percentage of viable remaining tumor.

### Adjuvant radiation therapy

External‐beam radiation was administered after surgical excision, typically starting 3–4 weeks postoperatively once wound healing permitted. Radiotherapy was performed using three‐dimensional conformal or intensity‐modulated radiotherapy techniques after CT simulation. The preoperative gross tumor volume was reconstructed based on clinical and pathologic information. Field sizes varied by tumor location and extent of surgery, generally adding 5 cm in craniocaudal directions and 1–2 cm in axial directions to create the clinical treatment volume. Expansions were limited by anatomic barriers, such as bone. The clinical treatment volume included the surgical scar, postsurgery changes visible on CT or magnetic resonance imaging, and drainage sites. An additional 0.5 cm to 1.0 cm margin was included in the planning target volume to account for setup variability, with the prescription dose covering the planning target volume. Patients were immobilized to allow optimal treatment angles, and bolus was added only if skin involvement was noted. When possible, weight‐bearing bones were spared from full‐dose irradiation, and portions of skin and soft tissues were spared to facilitate lymphatic rechanneling. Postoperative radiation therapy consisted of an initial dose of 45–50 grays (Gy), followed by a reduced‐volume boost of 14–16 Gy, for a total dose of 59.4–66.0 Gy. All treatments were delivered in daily fractions of 1.8–2.0 Gy, 5 days per week.

### Surveillance

During the surveillance phase, patients were monitored with a chest CT every 3 months for 2 years and then every 4 months for an additional 3 years, then a chest CT or chest x‐ray annually up to 10 years. Magnetic resonance imaging of the primary site was done every 4 months for 2 years, then every 6 months to 5 years.

### Statistical considerations

A power analysis conducted before study initiation indicated that 56 patients were needed to achieve 90% power for testing the hazard ratio (HR). The response rate to doxorubicin/ifosfamide–based chemotherapy regimens was estimated at 40%, with historical ranges between 26% and 66%.^22^, ^27^, ^28^ A responder on FDG‐PET was defined as having a ≥40% reduction in the SUVmax.^18^ Based on 5‐year disease‐free survival rates of 20% for nonresponders and 63% for responders (HR, 3.50)^18^ the sample size calculation required 56 patients for the 90% desired statistical power.

Kaplan–Meier estimates of PFS were compared between subgroups using log‐rank tests. Time was calculated as the number of months from diagnosis to disease progression, relapse, or death from any cause. Patients who were alive without progression or relapse were censored at the date of their last contact. Univariate analysis was used to identify factors predictive of PFS, and their independence was assessed using Cox regression. FDG‐PET metabolic response was defined based on SUVmax changes from baseline after one cycle (delta 1) or after all four chemotherapy cycles just before surgical excision (delta 2) on an intent‐to‐treat basis. A responder was defined as having an SUVmax reduction ≥40% from baseline. The SUVmax was also analyzed as a continuous variable.

Data were analyzed in two ways: first, as a cohort of all evaluable patients and, second, as a subgroup of patients with nonmetastatic disease at diagnosis who underwent primary tumor excision. Five patients who had metastatic disease at diagnosis and two patients who did not undergo primary tumor excision for unrelated, nononcologic reasons were excluded from the analysis.

## RESULTS

Sixty‐nine patients participated in the trial from May 2006 until May 2013 (Table 1). The median age was 54 years; five patients had locoregional metastasis at presentation, and 64 had localized disease. The range of histologies included was representative of typical high‐grade soft tissue sarcomas, consistent with the nomenclature used during the study enrollment period. In our cohort, 57 patients completed all four cycles of chemotherapy. Three patients received three cycles, six received two cycles, and three received only one cycle. Chemotherapy was discontinued early in 12 patients—five because of treatment‐related side effects and seven because of clear symptomatic disease progression, as determined by the primary investigators. Six patients did not undergo definitive surgical resection, and 52 of 63 patients who underwent surgical resection proceeded to receive adjuvant radiation therapy.

**TABLE 1:** Demographic characteristics, histology, and status at last follow‐up.

Variable, *n* = 69	No.	%
Age: Median [range], years	54 [20–79]	
Sex		
Men	42	61.0
Women	27	39.0
Locoregional metastasis		
No	64	92.0
Yes	5	7.0
Status at last follow‐up		
Alive, no progression	31	44.9
Alive, progression	10	14.5
Dead from sarcoma	26	37.7
Dead from other causes	2	1.9
Histology		
Undifferentiated sarcoma	42	61.0
Synovial sarcoma	15	22.0
Leiomyosarcoma	4	6.0
Liposarcoma	4	6.0
Fibrosarcoma	3	4.0
Malignant peripheral nerve sheath tumor	1	1.0

The follow‐up in this study was closed in May 2021. The median follow‐up time was 8.1 years (range 0.4–14.9 years). The median follow‐up time for living patients was 8.4 years (range, 2.8–14.9 years). Thirty‐four of 69 patients progressed or relapsed (49.3%), with a median progression time of 3.7 years (range, 0.1–14.7 years), and 28 of 69 died during follow‐up (42%), including 26 who died from sarcoma and two who died from other causes.

### Primary end point

#### Correlation of FDG‐PET response with PFS

**FIGURE 1:**
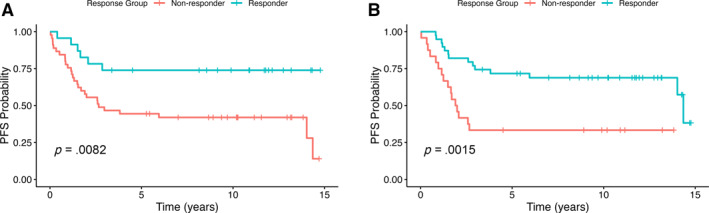
Kaplan–Meier plot of progression‐free survival. (A) FDG‐PET response after one cycle of chemotherapy [Delta 1]. (B) FDG‐PET response after four cycles of chemotherapy [Delta 2]. FDG‐PET indicates 5‐fluoro‐deoxyglucose–positron emission tomography.

When comparing FDG‐PET responders versus nonresponders, Kaplan–Meier analysis demonstrated a statistically significant difference in PFS as early as after one cycle of chemotherapy (delta 1). The 5‐year PFS rate for delta 1 responders was 74% (95% confidence interval [CI], 58%–94%), compared with 44% (95% CI, 32%–61%) in nonresponders. This difference remained significant at 10 years, with a PFS rate of 74% (95% CI, 58%–94%) in responders versus 42% (95% CI, 30%–59%) in nonresponders (log‐rank *p* = .0082; Figure 1A). In Cox regression analysis, delta 1 responders had a significantly reduced risk of progression or relapse compared with nonresponders (HR, 0.44; 95% CI, 0.21–0.90; *p* = .026).

Similarly, the PET response after completion of chemotherapy (delta 2) was also predictive of PFS. The 5‐year PFS rate for delta 2 responders was 72% (95% CI, 59%–87%) compared with 33% (95% CI, 19%–59%) in nonresponders. At 10 years, the PFS rate was 69% (95% CI, 56%–85%) in responders versus 33% (95% CI, 19%–59%) in nonresponders. This difference was statistically significant (log‐rank *p* = .0015; Figure 1B), and Cox regression confirmed a strong association between a delta 2 response and improved PFS (HR, 0.34; 95% CI, 0.17–0.68; *p* = .003).

### Secondary end points

#### Correlation of FDG‐PET response with OS

**FIGURE 2:**
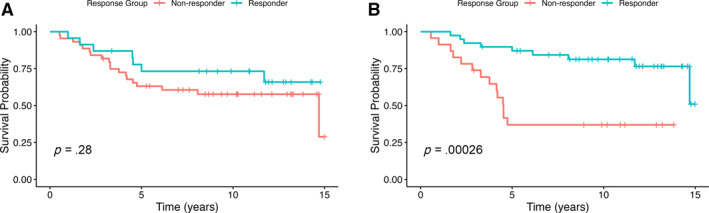
Kaplan–Meier plot of overall survival. (A) FDG‐PET response after one cycle of chemotherapy [Delta 1]. (B) FDG‐PET response after four cycles of chemotherapy [Delta 2]. FDG‐PET indicates 5‐fluoro‐deoxyglucose–positron emission tomography.

Based on Kaplan–Meier estimates, the 5‐year OS rate in delta 1 responders was 73% (95% CI, 57%–94%) compared with 63% (95% CI, 50%–79%) in nonresponders. At 10 years, the OS rate remained at 73% (95% CI, 57%–95%) in responders versus 58% (95% CI, 44%–75%) in nonresponders. The difference in survival between groups was not statistically significant (log‐rank *p* = .28; Figure 2A), which was consistent with Cox regression analysis (HR, 0.53; 95% CI, 0.22–1.25; *p* = .147).

In contrast, delta 2 responders had significantly improved outcomes. The 5‐year OS rate was 87% (95% CI, 77%–99%) in responders versus 37% (95% CI, 21%–64%) in nonresponders, and the 10‐year OS rate was 81% (95% CI, 70%–95%) versus 37% (95% CI, 21%–64%), respectively. This difference was statistically significant (log‐rank *p* = .00026; Figure 2B) and was confirmed by Cox regression analysis (HR, 0.20; 95% CI, 0.08–0.49; *p* < .001).

### Evolution of metabolic response during neoadjuvant treatment

FDG‐PET response analysis revealed that 23 of 69 patients (33.3%) achieved a decrease ≥40decrease in the SUVmax after one chemotherapy cycle (delta 1), and 39 of 64 patients (60.9%) achieved a decrease ≥40% in the SUVmax after four cycles of chemotherapy (delta 2). Some patients did not complete all four chemotherapy cycles. Therefore, there were fewer patients in the analysis of the multiple‐cycle response.

Among the 57 patients who completed four cycles of chemotherapy, 46 were not initially classified as responders at delta 1, and 23 of these patients subsequently met response criteria at delta 2. Of these 46 patients, eight had an increase in the SUVmax after cycle 1. Conversely, four patients who were classified as responders at delta 1 demonstrated disease progression at delta 2. Of these four patients, two were alive with no evidence of recurrence at last follow‐up, one was alive with progressive disease, and one died from sarcoma. In addition, one patient who was a responder at delta 1 did not complete all four cycles and thus was not evaluable at delta 2.

Of the 69 patients enrolled, 15 patients demonstrated progression or an increase in the SUVmax after one cycle of chemotherapy, with SUV changes ranging from +4.3% to +245%. Of these, 14 patients had an increase of at least 10%, and nine exhibited an increase of 20% or more. Despite early metabolic progression, eight patients subsequently met response criteria at delta 2, whereas two showed a decrease in FDG activity that did not reach the 40% threshold required to be classified as responders. The remaining five patients had confirmed progression at the completion of chemotherapy. The 5‐year PFS rate for this subgroup of patients who had progression at delta 1 was 46% (95% CI, 30%–74.3%), and the 5‐year OS rate was 65% (95% CI, 44%–95%).

### Multivariable regression of PFS, including FDG‐PET response (both initial and multiple cycle responses), with other prognostic variables

By using Cox regression analysis, we observed that a FDG‐PET response was independently and strongly predictive of PFS, whether analyzed as a continuous or categorical variable. Specifically, for every 10% decrease in the SUVmax after one cycle of chemotherapy (delta 1), the risk of progression or relapse decreased by 7%, corresponding to an HR of 0.93 (95% CI, 0.88–0.99; *p* = .015). This reduction in hazard reflects a meaningful protective effect associated with an early metabolic response. Similarly, a 10% decrease in the SUVmax in delta 1 was associated with improved OS, with an HR of 0.93 (95% CI, 0.87–1.00; *p* = .038).

A metabolic response after completion of chemotherapy (delta 2) was also significantly associated with improved outcomes. Each 10% reduction in the SUVmax at this timepoint was associated with a 7% lower risk of progression or relapse (HR, 0.93; 95% CI, 0.87–1.00; *p* = .037) and an 11% lower risk of death (HR, 0.89; 95% CI, 0.83–0.96; *p* = .003).

In additional analyses, PFS was not significantly associated with age, baseline tumor volume, American Joint Committee on Cancer stage at diagnosis, or completion of all four chemotherapy cycles.

### Correlation of FDG‐PET response to histologic response

Analysis of the histologic response to chemotherapy (defined as the percentage of nonviable tumor in the resected specimen) also revealed a correlation with a change in the SUVmax. For a delta 1 response, the correlation was 0.29 (*p* = .026; *n* = 59); and, for a delta 2 response, the correlation was 0.51 (*p* < .001; *n* = 52). The analysis confirmed that the more significant the decrease in the SUVmax after chemotherapy, the greater the percentage of nonviable tumor and the lower the percentage of remaining viable tumor cells in the tumor.

### Long‐term chemotherapy‐related toxicities

We observed that two patients developed acute renal failure requiring dialysis, resulting in chronic kidney disease. Importantly, five patients developed secondary malignancies later in life, consisting of: myelodysplastic syndrome (*n* = 2), and both died from the disease; breast cancer (*n* = 1), radiation‐induced chondrosarcoma (*n* = 1), and melanoma (*n* = 1). Finally, one patient developed autoimmune hemolytic anemia.

## DISCUSSION

This study represents one of the largest prospective trials, with the longest follow‐up time, of FDG‐PET for assessing the response to neoadjuvant chemotherapy in patients with high‐grade soft tissue sarcomas. Patients categorized as responders experienced a 20%–30% increase in 10‐year OS compared with nonresponders. Conventional CT scans have historically been insufficient for predicting sarcoma outcomes,^7^, ^8^, ^9^ underscoring the advantage of metabolic response by FDG‐PET.

Our data confirm that tumor metabolism on FDG‐PET more accurately reflects chemosensitivity than volumetric changes. Herrmann et al. demonstrated that a 25% decrease in the SUVmax after one cycle and a decrease >40% predicted 5‐year OS.^17^ Evilevitch et al. reported that a 60% decrease in the SUVmax was strongly correlated with OS.^16^ In our study, we used a 40% decrease, which proved prognostically meaningful and practical.

Multivariate analysis confirmed that an FDG‐PET response independently predicts PFS, with an HR of 0.93 per 10% decrease in the SUVmax, suggesting a dose–response relationship. Importantly, FDG‐PET after one cycle of chemotherapy provided early prognostic information, but nearly one half of eventual responders were identified only after four cycles of chemotherapy. In our cohort, 23 patients initially not deemed responders became responders at treatment completion, including some who had early SUVmax increases. Conversely, four early responders later progressed. These findings must be integrated into the broader clinical context. Apparent early progression may reflect transient flare or inflammation, and chemotherapy discontinuation should be approached cautiously given the possibility of a delayed response.

Long‐term toxicities were also observed in our cohort, including the development of secondary malignancies in five patients, three of which were clearly attributable to prior chemotherapy. This rate appears slightly higher than previously reported, underscoring the importance of a risk–benefit assessment when planning neoadjuvant chemotherapy, especially in patients with limited chemosensitivity.

In our study, we delivered four cycles of chemotherapy, and we did not see a metabolic response after one cycle in all patients who eventually achieved a metabolic response, suggesting that shorter regimens may be inadequate, even for metabolic responders. Whether additional consolidation beyond four cycles adds benefit remains unclear. The favorable toxicity profile of PLD and continuous‐infusion ifosfamide may have contributed to the good tolerability observed and better adherence, especially in older patients. Both PLD^21^, ^24^, ^29^ and ifosfamide administered as a continuous infusion^30^, ^31^ are known to have reduced toxicity compared with free doxorubicin plus ifosfamide delivered by intravenous push (infusion times of 1–2 hours) in multiple, divided doses.

Next‐generation sequencing may complement metabolic imaging by identifying actionable targets. In recent series, up to 47% of sarcomas harbored clinically relevant alterations.^32^, ^33^ The integration of PET with molecular profiling may refine risk‐adapted strategies. Results from the SARC032 trial (ClinicalTrials.gov identifier NCT03092323), in which pembrolizumab plus radiation improved OS in undifferentiated pleomorphic sarcoma and de‐differentiated liposarcoma, further suggest opportunities to combine metabolic imaging with novel therapeutics.^34^

The current study has several limitations, including the absence of standardized PET response criteria at study initiation^35^, ^36^ and the modest sample size. In addition, we did not formally assess changes using conventional CT scans, which limits the comparative analysis between imaging modalities. However, as discussed above, the response rate observed on conventional CT scans has never been shown to predict outcomes for patients with localized, high‐risk soft tissue sarcomas.

Three companion studies have been published from this trial examining blood‐based biomarkers and tumor biology.^37^, ^38^, ^39^ Together, these efforts reinforce FDG‐PET as a powerful tool for refining prognosis and informing therapy in high‐grade sarcomas.

## AUTHOR CONTRIBUTIONS

**Edward Y. Cheng**: Conceptualization, methodology, investigation, writing–original draft, writing–review and editing, supervision, resources, and funding acquisition. **Andrea P. Espejo Freire**: Data curation, formal analysis, writing–original draft, and writing–review and editing. **Juan C. Manivel**: Methodology and writing–review and editing. **Jerry W. Froelich**: Methodology and writing–review and editing. **Brenda J. Weigel**: Methodology and writing–review and editing. **Denis R. Clohisy**: Methodology and writing–review and editing. **Christian M. Ogilvie**: Methodology and writing–review and editing. **Rich Evans**: Formal analysis and writing–review and editing. **L. Chinsoo Cho**: Methodology and writing–review and editing. **Kathryn E. Dusenbery**: Methodology and writing–review and editing. **Keith M. Skubitz**: Conceptualization, methodology, data curation, investigation, writing–original draft, and writing–review and editing.

## CONFLICT OF INTEREST STATEMENT

The authors declared no conflicts of interest.

## Data Availability

The data that support the findings of this study are available on request from the corresponding author. The data are not publicly available because of privacy or ethical restrictions.

## References

[1] Frustaci S , Gherlinzoni F , De Paoli A , et al. Adjuvant chemotherapy for adult soft tissue sarcomas of the extremities and girdles: results of the Italian randomized cooperative trial. J Clin Oncol. 2001;19(5):1238‐1247. doi:10.1200/jco.2001.19.5.1238 11230464

[2] Tanaka K , Machida R , Kawai A , et al. Perioperative Adriamycin plus ifosfamide vs. gemcitabine plus docetaxel for high‐risk soft tissue sarcomas: randomised, phase II/III study JCOG1306. Br J Cancer. 2022;127(8):1487‐1496. doi:10.1038/s41416-022-01912-5 35871234 PMC9553903

[3] Tanaka K , Ozaki T . Adjuvant and neoadjuvant chemotherapy for soft tissue sarcomas: JCOG Bone and Soft Tissue Tumor Study Group. Jpn J Clin Oncol. 2021;51(2):180‐184. doi:10.1093/jjco/hyaa231 33313851

[4] Adjuvant chemotherapy for localised resectable soft‐tissue sarcoma of adults: meta‐analysis of individual data. Sarcoma Meta‐Analysis Collaboration. Lancet. 1997;350(9092):1647‐1654. 9400508

[5] Woll PJ , Reichardt P , Le Cesne A , et al. Adjuvant chemotherapy with doxorubicin, ifosfamide, and lenograstim for resected soft‐tissue sarcoma (EORTC 62931): a multicentre randomised controlled trial. Lancet Oncol. 2012;13(10):1045‐1054. doi:10.1016/s1470-2045(12)70346-7 22954508

[6] Benjamin RS , Choi H , Macapinlac HA , et al. We should desist using RECIST, at least in GIST. J Clin Oncol. 2007;25(13):1760‐1764. doi:10.1200/jco.2006.07.3411 17470866

[7] Choi H , Charnsangavej C , Faria SC , et al. Correlation of computed tomography and positron emission tomography in patients with metastatic gastrointestinal stromal tumor treated at a single institution with imatinib mesylate: proposal of new computed tomography response criteria. J Clin Oncol. 2007;25(13):1753‐1759. doi:10.1200/jco.2006.07.3049 17470865

[8] Choi H. Role of imaging in response assessment and individualised treatment for sarcomas. Clin Oncol (R Coll Radiol). 2017;29(8):481‐488. 10.1016/j.clon.2017.04.002 28506521 10.1016/j.clon.2017.04.002

[9] Stacchiotti S , Collini P , Messina A , et al. High‐grade soft‐tissue sarcomas: tumor response assessment—pilot study to assess the correlation between radiologic and pathologic response by using RECIST and Choi criteria. Radiology. 2009;251(2):447‐456. doi:10.1148/radiol.2512081403 19261927

[10] Stacchiotti S , Verderio P , Messina A , et al. Tumor response assessment by modified Choi criteria in localized high‐risk soft tissue sarcoma treated with chemotherapy. Cancer. 2012;118(23):5857‐5866. doi:10.1002/cncr.27624 22605504

[11] Schulte M , Brecht‐Krauss D , Heymer B , et al. Fluorodeoxyglucose positron emission tomography of soft tissue tumours: is a non‐invasive determination of biological activity possible? Eur J Nucl Med. 1999;26(6):599‐605. doi:10.1007/s002590050427 10369945

[12] Partovi S , Chalian M , Fergus N , et al. Magnetic resonance/positron emission tomography (MR/PET) oncologic applications: bone and soft tissue sarcoma. Semin Roentgenol. 2014;49(4):345‐352. doi:10.1053/j.ro.2014.04.004 25498231

[13] Benz MR , Dry SM , Eilber FC , et al. Correlation between glycolytic phenotype and tumor grade in soft‐tissue sarcomas by 18F‐FDG PET. J Nucl Med. 2010;51(8):1174‐1181. doi:10.2967/jnumed.109.074229 20660389 PMC3197812

[14] Völker T , Denecke T , Steffen I , et al. Positron emission tomography for staging of pediatric sarcoma patients: results of a prospective multicenter trial. J Clin Oncol. 2025;25(34):5435‐5441. doi:10.1200/JCO.2007.12.2473 18048826

[15] Benz MR , Czernin J , Allen‐Auerbach MS , et al. FDG‐PET/CT imaging predicts histopathologic treatment responses after the initial cycle of neoadjuvant chemotherapy in high‐grade soft‐tissue sarcomas. Clin Cancer Res. 2009;15(8):2856‐2863. doi:10.1158/1078-0432.ccr-08-2537 19351756 PMC4068269

[16] Evilevitch V , Weber WA , Tap WD , et al. Reduction of glucose metabolic activity is more accurate than change in size at predicting histopathologic response to neoadjuvant therapy in high‐grade soft‐tissue sarcomas. Clin Cancer Res. 2008;14(3):715‐720. doi:10.1158/1078-0432.ccr-07-1762 18245531

[17] Herrmann K , Benz MR , Czernin J , et al. 18F‐FDG‐PET/CT imaging as an early survival predictor in patients with primary high‐grade soft tissue sarcomas undergoing neoadjuvant therapy. Clin Cancer Res. 2012;18(7):2024‐2031. doi:10.1158/1078-0432.ccr-11-2139 22338012 PMC3431618

[18] Schuetze SM , Rubin BP , Vernon C , et al. Use of positron emission tomography in localized extremity soft tissue sarcoma treated with neoadjuvant chemotherapy. Cancer. 2005;103(2):339‐348. doi:10.1002/cncr.20769 15578712

[19] Kubo T , Furuta T , Johan MP , Ochi M . Prognostic significance of 18F‐FDG PET at diagnosis in patients with soft tissue sarcoma and bone sarcoma; systematic review and meta‐analysis. Eur J Cancer. 2016;58:104‐111. doi:10.1016/j.ejca.2016.02.007 26990930

[20] Skubitz KM , Hamdan H , Thompson RC Jr . Ambulatory continuous infusion ifosfamide with oral etoposide in advanced sarcomas. Cancer. 1993;72(10):2963‐2969. doi:10.1002/1097-0142(19931115)72:10<2963::aid-cncr2820721017>3.0.co;2-w 8221562

[21] Judson I , Radford JA , Harris M , et al. Randomised phase II trial of pegylated liposomal doxorubicin (DOXIL®/CAELYX®) versus doxorubicin in the treatment of advanced or metastatic soft tissue sarcoma: a study by the EORTC Soft Tissue and Bone Sarcoma Group. Eur J Cancer. 2001;37(7):870‐877. doi:10.1016/s0959-8049(01)00050-8 11313175

[22] Liu X , Jiang S , Wang H , et al. Pegylated liposomal doxorubicin combined with ifosfamide for treating advanced or metastatic soft‐tissue sarcoma: a prospective, single‐arm phase II study. Clin Cancer Res. 2022;28(24):5280‐5289. doi:10.1158/1078-0432.ccr-22-1785 36239473

[23] Savani M , Murugan P , Skubitz KM . Long‐term cure of soft tissue sarcoma with pegylated‐liposomal doxorubicin after doxorubicin and ifosfamide failure. Clin Sarcoma Res. 2019;9(1):1. doi:10.1186/s13569-018-0111-0 30651969 PMC6332634

[24] Skubitz KM . Phase II trial of pegylated‐liposomal doxorubicin (Doxil™) in sarcoma. Cancer Invest. 2003;21(2):167‐176. doi:10.1081/cnv-120016412 12743981

[25] Coindre JM . Grading of soft tissue sarcomas: review and update. Arch Pathol Lab Med. 2006;130(10):1448‐1453. doi:10.5858/2006-130-1448-gostsr 17090186

[26] Costa J , Wesley RA , Glatstein E , Rosenbergs SA . The grading of soft tissue sarcomas. Results of a clinicohistopathologic correlation in a series of 163 cases. Cancer. 1984;53(3):530‐541. doi:10.1002/1097-0142(19840201)53:3<530::aid-cncr2820530327>3.0.co;2-d 6692258

[27] Judson I , Verweij J , Gelderblom H , et al. Doxorubicin alone versus intensified doxorubicin plus ifosfamide for first‐line treatment of advanced or metastatic soft‐tissue sarcoma: a randomised controlled phase 3 trial. Lancet Oncol. 2014;15(4):415‐423. doi:10.1016/s1470-2045(14)70063-4 24618336

[28] Patel SR , Vadhan‐Raj S , Burgess MA , et al. Results of two consecutive trials of dose‐intensive chemotherapy with doxorubicin and ifosfamide in patients with sarcomas. Am J Clin Oncol. 1998;21(3):317‐321. doi:10.1097/00000421-199806000-00025 9626808

[29] Lyass O , Uziely B , Ben‐Yosef R , et al. Correlation of toxicity with pharmacokinetics of pegylated liposomal doxorubicin (Doxil) in metastatic breast carcinoma. Cancer. 2000;89(5):1037‐1047. doi:10.1002/1097-0142(20000901)89:5<1037::aid-cncr13>3.0.co;2-z 10964334

[30] Sanfilippo R , Bertulli R , Marrari A , et al. High‐dose continuous‐infusion ifosfamide in advanced well‐differentiated/dedifferentiated liposarcoma. Clin Sarcoma Res. 2014;4(1):16. doi:10.1186/2045-3329-4-16 25628856 PMC4307996

[31] Plutt AA , Stanton MP , Zembillas AS , Pierson CE , Zahler S , Anderson PM . Excellent tolerability of ifosfamide and mesna via continuous infusion in a pediatric patient population. J Pediatr Hematol Oncol. 2022;44(8):e1029‐e1032. doi:10.1097/mph.0000000000002361 34862353

[32] Cote GM , He J , Choy E . Next‐generation sequencing for patients with sarcoma: a single center experience. Oncologist. 2018;23(2):234‐242. doi:10.1634/theoncologist.2017-0290 28860410 PMC5813739

[33] Gusho CA , Weiss MC , Lee L , et al. The clinical utility of next‐generation sequencing for bone and soft tissue sarcoma. Acta Oncol. 2022;61(1):38‐44. doi:10.1080/0284186x.2021.1992009 34686105

[34] Mowery YM , Ballman KV , Hong AM , et al. Safety and efficacy of pembrolizumab, radiation therapy, and surgery versus radiation therapy and surgery for stage III soft tissue sarcoma of the extremity (SU2C‐SARC032): an open‐label, randomised clinical trial. Lancet. 2024;404(10467):2053‐2064. doi:10.1016/s0140-6736(24)01812-9 39547252 PMC11842127

[35] Wahl RL , Jacene H , Kasamon Y , Lodge MA . From RECIST to PERCIST: evolving considerations for PET response criteria in solid tumors. J Nucl Med. 2009;50(suppl 1):122S‐150S. doi:10.2967/jnumed.108.057307 19403881 PMC2755245

[36] Young H , Baum R , Cremerius U , et al. Measurement of clinical and subclinical tumour response using [^18^F]‐fluorodeoxyglucose and positron emission tomography: review and 1999 EORTC recommendations. European Organization for Research and Cancer (EORTC) PET Study Group. Eur J Cancer. 1999;35(13):1773‐1782. doi:10.1016/S0959-8049(99)00229-4 10673991

[37] Skubitz KM , Domingo‐Musibay E , Lindgren BR , Cheng EY . Prospective trial of neutrophil/lymphocyte ratio and other blood counts as biomarkers of survival among patients with high‐grade soft tissue sarcomas treated with pegylated liposomal doxorubicin and ifosfamide. Cancers (Basel). 2022;14(14):3419. doi:10.3390/cancers14143419 35884480 PMC9316699

[38] Skubitz KM , Lindgren BR , Domingo‐Musibay E , Cheng EY . Prospective trial of monocyte count as a biomarker of hand‐foot syndrome among patients with soft tissue sarcomas treated with pegylated liposomal doxorubicin and ifosfamide. Cureus. 2022;14(4):e24498. doi:10.7759/cureus.24498 35651410 PMC9135613

[39] Skubitz KM , Wilson JD , Cheng EY , Lindgren BR , Boylan KLM , Skubitz APN . Effect of chemotherapy on cancer stem cells and tumor‐associated macrophages in a prospective study of preoperative chemotherapy in soft tissue sarcoma. J Transl Med. 2019;17(1):130. doi:10.1186/s12967-019-1883-6 30999901 PMC6471853

